# Predicting non-isometric fatigue induced by electrical stimulation pulse trains as a function of pulse duration

**DOI:** 10.1186/1743-0003-10-13

**Published:** 2013-02-02

**Authors:** M Susan Marion, Anthony S Wexler, Maury L Hull

**Affiliations:** 1 Biomedical Engineering Program, University of California, Davis, CA, 95616, USA; 2 Mechanical and Aerospace Engineering, University of California, Davis, CA, 95616, USA

**Keywords:** Functional electrical stimulation (FES), Non-isometric, Muscle fatigue, Mathematical model, Pulse duration

## Abstract

**Background:**

Our previous model of the non-isometric muscle fatigue that occurs during repetitive functional electrical stimulation included models of force, motion, and fatigue and accounted for applied load but not stimulation pulse duration. Our objectives were to: 1) further develop, 2) validate, and 3) present outcome measures for a non-isometric fatigue model that can predict the effect of a range of pulse durations on muscle fatigue.

**Methods:**

A computer-controlled stimulator sent electrical pulses to electrodes on the thighs of 25 able-bodied human subjects. Isometric and non-isometric non-fatiguing and fatiguing knee torques and/or angles were measured. Pulse duration (170–600 μs) was the independent variable. Measurements were divided into parameter identification and model validation subsets.

**Results:**

The fatigue model was simplified by removing two of three non-isometric parameters. The third remained a function of other model parameters. Between 66% and 77% of the variability in the angle measurements was explained by the new model.

**Conclusion:**

Muscle fatigue in response to different stimulation pulse durations can be predicted during non-isometric repetitive contractions.

## Background

Functional Electrical Stimulation (FES) protocols use combinations of stimulation parameters (train duration, interpulse interval, pulse duration, and pulse amplitude) to produce functional movements in individuals with paralysis due to stroke or spinal cord injury (SCI). Unlike physiologically induced neuromuscular activation, FES synchronously activates motor units according to their current thresholds relative to the local extracellular current which is dependent on the distance from the electrodes [1,2]. Consequently, the recruitment of motor units is random [3,4] as compared to the order followed by the central nervous system, which recruits the smaller, fatigue-resistant motor units first and the larger, more fatigable motor units last. When the motor units are activated synchronously the body cannot derecruit motor units as they fatigue and recruit new fresh motor units to replace them [5]. This random recruitment order together with synchronous activation are thought to be two of the major causes for excessive muscle fatigue during FES.

A mathematical model capable of predicting angular excursion, angular velocity, and joint torque during fatiguing contractions as a function of the stimulation parameters could be used to mathematically test combinations of independent and dependent variables to identify stimulation strategies that minimize fatigue. In addition, a validated model could predict force during fatiguing contractions in situations where force cannot be measured easily, such as during general non-isometric leg extensions. The term non-isometric indicates that the joint angle and thus the length of the musculo-tendon unit continually changes as the muscle contracts and relaxes. The phrase general non-isometric indicates that the leg is free to move solely in response to muscle forces. Although many models of non-isometric non-fatiguing contractions [6-8] and isometric fatiguing contractions [9-14] have been developed, only two models of non-isometric fatiguing contractions in humans appear in the literature [15,16]. The model by Marion and colleagues [16] is the only one that has been experimentally validated to predict non-isometric fatigue in response to electrical stimulation.

In our previous study [16] we were interested in predicting non-isometric fatigue when the tension per activated motor unit was increased through the application of external loads. A similar situation may occur, for instance, in the spinal cord injured population when the relative resistive torque at the knee as compared to the number of activated motor units in the quadriceps increases as atrophy progresses. We are now interested in determining whether our non-isometric fatigue model can predict angular excursion, angular velocity, and joint torque due to stimulation of the quadriceps muscles at different pulse durations. This interest stems from the following reasons: 1) previous studies suggest that torque output can be predictably controlled and fatigue minimized by simultaneously controlling stimulation pulse duration and frequency during repetitive electrical stimulation [17-19], 2) others have shown the effect of pulse frequency on isometric fatigue and suggest that frequency should be minimized [20,21], 3) our isometric force-fatigue model accounts for pulse frequency and pattern [10,20], but neither the isometric nor the non-isometric force-fatigue model account for pulse duration, 4) studies suggest that relative isometric fatigue (compared to the initial torque) does not change with pulse duration [4,21], therefore pulse duration can be increased to maintain torque, and 5) the relationship between pulse duration and non-isometric fatigue has not been reported, therefore it is unknown whether pulse duration can be increased to maintain torque and/or excursion. Because the overall objective of an ideal FES pulse train is to obtain the desired force and motion while minimizing fatigue, a fatigue model that takes pulse duration into account is required.

The objectives of this study were to: 1) further develop our model of FES non-isometric fatigue to take into account pulse duration, while simultaneously minimizing the number of parameter identification sessions with subjects by minimizing the number of model parameters, 2) experimentally validate the model at different pulse durations, and 3) present outcome measures, such as predicted angular excursion, angular velocity, joint torque, and power (torque (N-m) x angular velocity (rad/s)) due to stimulation, that can be compared over time for different independent variables. For consistency with our previous study, we chose general non-isometric leg extensions to further develop our model of non-isometric muscle fatigue. For reference, the term leg is defined as that section of the lower limb between the knee and ankle.

## Methods

### Mathematical model

The force-motion-fatigue model developed by Ding and colleagues [7,10,16] was used for this study (see Table 1 for definitions of symbols). The force-motion model [7,16] describes muscle activation, contraction dynamics, the force-angle relationship, and the force-angular velocity relationship. The input is the time the pulses are delivered, and the output is the force (*F*) at the ankle predicted for each time point (see Appendix).

(1) $$\frac{dC_{N}}{\mathrm{dt}}=\frac{1}{\tau_{c}}\sum_{i=1}^{n}R_{i}\exp\left(-\frac{t-t_{i}}{\tau_{c}}\right)-\frac{C_{N}}{\tau_{c}}$$

(1a) $$R_{i}=\{\begin{array}{c} 1\\ 1+\left(R_{0}-1\right)\exp\left(-\frac{t_{i}-t_{i-1}}{\tau_{c}}\right) \end{array}\begin{array}{ccc} & i=1 & \\ & i>1 & \end{array}$$

(2) $$\frac{\mathrm{dF}}{\mathrm{dt}}=\left[G+A\right]\frac{C_{N}}{K_{m}+C_{N}}-\frac{F}{\tau_{1}+\tau_{2}\frac{C_{N}}{K_{m}+C_{N}}}$$

(2a) $$A=A_{90}\left(a{\left(90-\theta \right)}^{2}+b\left(90-\theta \right)+1\right)$$

(2b) $$G=V_{1}\theta \exp\left(-V_{2}\theta \right)\frac{\mathrm{d\theta}}{\mathrm{dt}}$$

(3) $$\frac{d^{2}\theta }{dt^{2}}=\frac{L}{I}\left[\left(F_{\mathrm{load}}+F_{M}\right)\cos\left(\theta +\lambda \right)-F\right]$$

**Table 1:** Definition of symbols and acronyms

**Term**	**Unit**	**Definition**
A_90_	N ms^-1^	Scaling factor reflecting magnitude of force at 90°
a	deg^-2^	Defines parabolic shape of ankle force - knee angle relationship
α_A_	ms^-2^	Force scaling factor in fatigue model for force-motion model parameter A
α_Km_	ms^-1^N^-1^	Force scaling factor in fatigue model for force-motion model parameter K_m_
α_τ1_	N^-1^	Force scaling factor in fatigue model for force-motion model parameter τ_1_
b	deg^-1^	Defines parabolic shape of ankle force-knee angle relationship
β_A_	ms^-1^deg^-1^	Angular velocity x force scaling factor in fatigue model for force-motion model parameter A
β_Km_	deg^-1^N^-1^	Angular velocity x force scaling factor in fatigue model for force-motion model parameter K_m_
β_τ1_	ms deg^-1^N^-1^	Angular velocity x force scaling factor in fatigue model for force-motion model parameter τ_1_
CFT	-	Constant frequency train
C_N_	-	Normalized concentration of Ca^2+^-troponin complex
F	N	Instantaneous force near the ankle due to stimulation
F_load_	N	Load applied at ankle during general non-isometric leg extensions
F_M_	N	Represents the resistance to knee extension due to the weight of the leg and all other passive resistance about the knee joint
TTI	N s	Torque Time Integral
I	kg m^2^	Net mass moment of inertia of the leg plus the applied load
K_m_	-	Similar to Michaelis-Menten constant. Affinity of actin strong binding site for myosin
L	m	Effective moment arm from knee joint center of rotation to resultant force vector near ankle
λ	deg	90° minus the knee flexion angle of the resting non-isometric leg
n	-	Number of stimuli in train before time t
R_0_	-	Characterizes the magnitude of enhancement in C_N_ from the following stimuli
R_i_	-	Accounts for differences in activation for each pulse relative to first pulse of train
SCI	-	Spinal Cord Injury
t_i_	ms	Time of the i^th^ stimulation
τ_1_	ms	Time constant of force decline in the absence of strongly bound cross-bridges
τ_2_	ms	Time constant of force decline due to actin-myosin friction in cross-bridges
τ_c_	ms	Time constant controlling the rise and decay of C_N_
τ_fat_	ms	Time constant for force-motion model parameters A, K_m1_, and τ_1_ during fatigue
θ	deg	Knee flexion angle, where full extension was 0˚
V_1_	N deg^-2^	Scaling factor in the term G
V_2_	deg^-1^	Constant
VFT	-	Variable frequency train

Equation 1 models the rate-limiting step that leads to the formation of strongly bound crossbridges, and it represents the activation dynamics. Equation 2 describes the generation of the instantaneous force (*F*) near the ankle due to stimulation. It was derived from a Maxwell model of linear viscoelasticity in series with a motor [22]. The terms *A* and *G* represent the torque-angle [23] and torque-angular velocity [8] relationships, respectively. A torque-pulse duration relationship has not been derived yet for this force-motion model of non-isometric leg extensions. To meet the overall objective of the current study, to predict the effect of a range of pulse durations on muscle fatigue, the initial non-fatigue torque was measured at the pulse duration of interest just prior to the fatigue test. The Michaelis-Menten term, *C*_*N*_/(*K*_*m*_ + *C*_*N*_), scaled by *A* and *G*, drives the development of force. The last term in Equation (2) accounts for the force decay over two time constants, *τ*_*1*_ and *τ*_*2*_. Equation 3 models the dynamics of the leg distal to the knee. The term *F*_*M*_ represents the resistance to knee extension due to the weight of the leg and all other passive resistance about the knee joint, whereas *F*_*load*_ is the load applied at the ankle (e.g. 4.54 kg; see Appendix). The term *λ* is added to the angle at the knee to ensure that angular acceleration is zero at the beginning of stimulation. Often the resting knee angle is not exactly 90°, *λ* is the difference.

The fatigue model [10,16] monitors changes in the three force-motion model parameters that change with fatigue, *A*_*90*_, *K*_*m*_ and *τ*_*1*_. For each time step the input is instantaneous force (Equation 2) and angular velocity (from angular acceleration in Equation 3) from the force-motion model (once all force and fatigue model parameters have been identified) for that time step. The output is the *A*_*90*_*, K*_*m*_ and *τ*_*1*_ to be used in the force-motion model at the next time step.

(4) $$\frac{dA_{90}}{\mathrm{dt}}=-\frac{A_{90}-A_{90,0}}{\tau_{\mathrm{fat}}}+\left(\alpha_{A}+\beta_{A}\frac{\mathrm{d\theta}}{\mathrm{dt}}\right)F$$

(5) $$K_{m}=K_{m1}+K_{m2}$$

(6) $$\frac{dK_{m1}}{\mathrm{dt}}=-\frac{K_{m1}-K_{m1,0}}{\tau_{\mathrm{fat}}}+\left(\alpha_{\mathrm{Km}}+\beta_{\mathrm{Km}}\frac{\mathrm{d\theta}}{\mathrm{dt}}\right)F$$

(7) $$\frac{dK_{m2}}{\mathrm{dt}}=-\alpha_{\mathrm{Km}}F$$

(8) $$\frac{d\tau_{1}}{\mathrm{dt}}=-\frac{\tau_{1}-\tau_{1,0}}{\tau_{\mathrm{fat}}}+\left(\alpha_{\tau_{1}}+\beta_{\tau_{1}}\frac{\mathrm{d\theta}}{\mathrm{dt}}\right)F$$

The time constant *τ*_*fat*_ characterizes the rate of change of parameters *A*_*90*_*, K*_*m1*_*,* and *τ*_*1*_ from the pre-fatigue values (*A*_*90,0*_*, K*_*m1,0,*_ and *τ*_*1,0*_) to that in a steady state of fatigue. All of these terms have been reported previously [10,16,24] and the same procedures were used here to identify the values.

### Parameter identification

**Figure 1:**
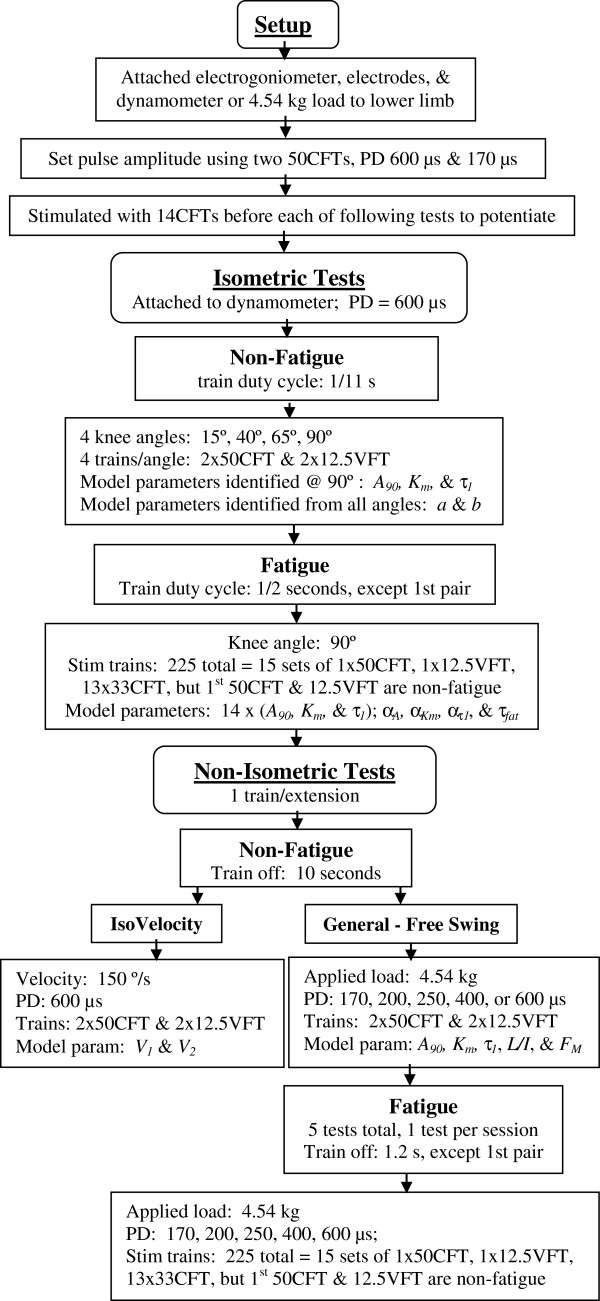
**Flow chart of the testing protocol and the model parameters identified from each test.** The train duty cycle for the general non-isometric contractions varied with pulse duration (PD; see results). Parameter *τ*_*1*_ was identified separately using the force at the end of each contraction. Parameters *a* and *b* were identified by fitting parameter *A* predicted by Equation (2a) to parameter *A* from Equation (2) for all four knee angles. Fitting *A*_*90*_*, K*_*m*_*,* and *τ*_*1*_ predicted by the fatigue model to *A*_*90*_*, K*_*m*_*,* and *τ*_*1*_ from the force model for the isometric pre-fatigue and isometric fatiguing contractions identified the fatigue model parameters *α*_*A*_*, α*_*Km*_*, α*_*τ1*_*,* and *τ*_*fat*_ (Equations 4–8). Initially, parameter *β*_*τ1*_ (Equation 8) was identified by fitting model predicted angles and angular velocities to the measurements collected at 170, 200, and 600 μs during the fatiguing leg extensions. An equation for *β*_*τ1*_ (Equation 9) was then derived from correlations with the parameters in the model.

**Figure 2:**
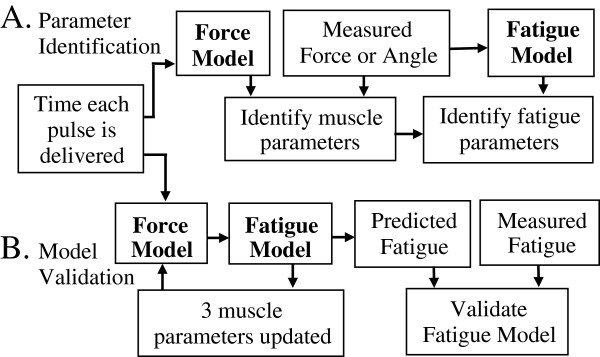
**Block diagram demonstrating parameter identification (A) and prediction of fatigue (B) using the force-motion (Force) and fatigue models.** During parameter identification (**A**) force-motion model parameters (muscle parameters) were identified by fitting the modeled forces, angles, and angular velocities to the measurements collected prior to and during the fatiguing protocol in response to the 50CFT and 12.5VFT trains. The fatigue model parameters were identified by fitting the parameters *A*_*90*_, *K*_*m*_, and *τ*_*1*_ (3 of the 15 muscle parameters) derived from the force-motion model to the parameters *A*_*90*_, *K*_*m*_, and *τ*_*1*_ predicted by the fatigue model. During model validation (**B**), the force and velocity predicted by the force-motion model enter the fatigue model at a given time step. The fatigue model predicts the parameters *A*_*90*_, *K*_*m*_, and *τ*_*1*_ to be used by the force-motion model for the next time step. Upon completion of all time steps the predicted fatigue is compared to the measured fatigue to validate the model.

The force-motion-fatigue model contains a total of nineteen parameters. Parameters *R*_0_ and *τ*_*c*_ were held constant at 2 (unitless) [10] and 20 ms [20], respectively (see citations for results showing the derivation of these values). Fourteen of the remaining parameters, *A*_*90*_*, a, b, K*_*m*_*, τ*_*1*_, *τ*_*2*_*, V*_*1*_*, V*_*2*_*, L/I,* and *F*_*M,*_ from the force-motion model and *α*_*A*_*, α*_*Km*_*, α*_*τ1*_, and *τ*_*fat*_ from the fatigue model, required identification to both develop and validate the model, as well as to generate predictions. These parameters were identified from leg extension measurements, first from the development then from the validation groups of subjects (see Experimental Procedures and Figures 1 and 2). The remaining fatigue model parameters, *β*_*A*_*, β*_*Km*_*,* and *β*_*τ1*_, were initially identified from measurements and only from the development subjects. Model parameters were identified through minimization of the sum of squares error between the measured and modeled values via a Particle Swarm Optimization algorithm [25] followed by a nonlinear least-squares algorithm (MatLab®) [26]. Optimizations were repeated several times to confirm that solutions had converged to the “global” minimum.

Preliminary results showed that for many subjects none of the three *β* parameters were needed for accurate predictions of the measured angles and angular velocities. After careful examination of the subjects that required *β*, we discovered that only *β*_*τ1*_ was necessary to predict fatigue in those subjects. Thus, although *β*_*A*_ and *β*_*Km*_ were employed in previous work [16], we postulated that *β*_*A*_ and *β*_*Km*_ were not necessary for modeling non-isometric fatigue; we explored this hypothesis as described in the Results.

### Experimental procedures

#### Equipment and participant setup

Twenty-five healthy subjects, 14 men and 11 women (ages 21–48), with no history of lower extremity orthopedic problems voluntarily participated in this study and signed informed consent agreements. This study was approved by the University of California Human Subjects Review Board. Data from 5 men and 5 women (ages 19–25) from the previous study on predicting fatigue at different loads [16] were also analyzed to further validate the model.

The experimental setup was similar to that described previously [16,27] (Figure 1). Subjects were seated in a backward-inclined (15° from vertical) chair of an exercise dynamometer (System 2, Biodex Medical Systems, Inc., Shirley, New York). The trunk, hips, and thigh were strapped to the chair, thus fixing the hip angle and limiting leg movement. The ankle was strapped to the lever arm of the dynamometer for the isometric and isovelocity tests. The axis of rotation of the knee joint was aligned with the axis of rotation of the dynamometer. A custom built electrogoniometer with two potentiometers, one positioned at the hip and the other at the knee axis of rotation, was strapped to the lower limb and trunk to measure joint angles. Customized software (LabView 8.0, National Instruments Corporation, Austin, TX) collected the digitized voltage signals at 300 Hz from the dynamometer torque transducer and the electrogoniometer. Two 7.5 cm × 12.5 cm self-adhesive stimulating electrodes (WF35 from http://www.tensproducts.com) were placed on the skin of the right thigh, one at the proximal and the other at the distal end of the quadriceps muscles. The electrode positions were adjusted until both a maximum amplitude and a constant shape of the torque-time curve were achieved at 4 different knee angles, 90°, 65°, 40° and 20° (where full extension was 0˚) and at 3 of the 5 pulse durations to be tested (min, mid, and max) and until the amount of non-planar movement of the leg during general non-isometric leg extensions was minimized. In some subjects this resulted in the anode being positioned proximal to the cathode.

Customized software controlled the rate that monophasic pulses were delivered by the Grass S48 stimulator (Grass Technologies, Astro-Med, Inc. Product Group, West Warwick, RI) to the electrodes. A constant-voltage transcutaneous system was used to minimize the risk of high current densities that can occur with constant-current systems if electrode contact with the skin is reduced. Others, also studying pulse duration, have used a similar system [4,28]. Stimulus efficacy may have changed with increasing muscle contraction during delivery of the train because the tissues under the skin can move relative to the electrodes as the leg extends and because the current was not held constant, i.e. maximum stimulation of excitable tissue frequently occurs at the beginning of the pulse when current is maximum [29,30]. Stimulus efficacy also may have changed over time during delivery of repetitive fatiguing trains of pulses because of sweating, which reduces skin impedance, and because of increased blood flow due to increased tissue temperature [31]. However, the parameters for the force-motion-fatigue model are identified from experimental measurements from each subject, therefore the model can and does account for each subject’s muscle response to the stimulation system used for the measurements. An attached SIU8T stimulus isolation unit (Grass Technologies) isolated the electrodes from ground, providing greater safety to the subject.

#### Testing sessions – general information common to All tests

Each subject participated in 4 to 6 testing sessions. Thirteen subjects were used for model development; the remaining 12 for model validation. Subjects were asked to refrain from strenuous exercise 24 hours prior to each testing session. Successive sessions were separated by at least 48 hours to allow the muscles to recover and again yield the maximum torque measured by the dynamometer prior to fatigue. Prior to, within the consent to participate form, and during the testing sessions, participants were asked to relax their legs so that the stimulation trains could be applied to relaxed quadriceps femoris muscles. The consent form states that the sessions may have to be repeated if they are unable to fully relax their leg. Torque and knee angle were monitored in real-time during the tests. The traces for each had consistent shapes appearing at timed intervals (during electrical stimulation). Volitional activation could be detected easily as alterations to the regularity/uniformity of the traces. Additionally, volitional activation during general non-isometric contractions prevents or alters the pendulum motion of the leg that occurs immediately after the leg drops to the resting position after cessation of a stimulation train. If the real-time traces on the plots or the pendulum motion of the leg looked unusual the test was stopped and the subject was gently reminded to relax.

The stimulation amplitude was set to produce maximum excursion of the freely swinging leg for both the minimum and maximum pulse durations while a 4.54 kg load was strapped to the ankle. This load was applied during all general non-isometric tests. Ten pounds or 4.54 kg was chosen for two reasons: 1) because it was used in previous studies to develop the force-motion model used in the current study and to identify its parameters [7,16] and 2) because our previous study [16] and pilot measurements suggested this load would provide measureable declines in force for the desired range of pulse durations during the fatiguing contractions. The stimulation amplitude was set at the voltage that extended the leg to ~15° with two 50 Hz trains, one with 600 μs pulses, trains no shorter than 0.2 seconds, and the other with 170 μs pulses, trains no longer than 0.8 seconds. This assured a maximum range of motion for every subject at all pulse durations, and thus a maximum range of fatigue for model development. When using a Grass stimulator quadriceps force approaches steady state near a pulse duration of 600 μs [4,32], therefore 600 μs was selected as the maximum pulse duration. The minimum train duration was set at 0.2 seconds so that at least two pulses would be delivered at the lowest frequency tested. Pulse durations shorter than 170 μs were not used because the target excursion could not be reached at shorter durations by all subjects at the maximum pulse amplitude that was limited by the 0.2 second train of 600 μs pulses. Increasing the train duration longer than 0.8 seconds with 170 μs pulses did not increase the excursion of the leg. The pulse amplitude depended on the subject, ranging from 30 to 83 volts.

Both constant (CFT) and variable (VFT) frequency trains, containing equally spaced singlet pulses or an initial doublet (5 ms between pulses within the doublet) followed by equally spaced singlet pulses, respectively, were applied. Previous studies [10] have shown these two types of trains to be effective for identifying the model parameters, in particular 50CFT-12.5VFT pairs, where 50 and 12.5 refer to the frequency (Hz) of the singlet pulses. At the beginning of every test, the quadriceps were held isometric and stimulated with twelve 14CFTs (14 Hz pulse frequency), with 0.8 second train durations and 5 seconds between trains, to potentiate the muscle [23]. Twitch responses initially increase during repeated low-frequency stimulation (staircase phenomenon or twitch potentiation) and after a tetanic contraction (post-tetanic potentiation) [33]. The mechanism of force enhancement may be related to phosphorylation of myosin light chains and increased Ca^2+^ sensitivity [34].

#### Isometric tests

##### Non-fatigue isometric

Parameters *A*_*90*_*, Km, τ*_*1*_, *τ*_*2*_*, a,* and *b* were identified from the non-fatiguing isometric contractions from one testing session. Torque in response to two pairs of testing trains (2 × 50CFT-12.5VFT pair) was measured at each of 4 knee angles (15°, 40°, 65°, 90°). The order of the angles varied from session to session and subject to subject. The pulse duration was 600 μs, train duration was 1 second, and the rest between trains was 10 seconds. The muscles rested 4 minutes between angles, which was sufficient because the duty cycle and the number of trains delivered were too low to fatigue the muscles [10]. Measured forces were compared to modeled forces for initial identification of *A*_*90*_*, K*_*m*_*, τ*_*1*_, and *τ*_*2*_. Parameter *A* identified from the force-motion model (Equation 2) and parameter *A* predicted by the parabolic equation were compared to identify *a* and *b* (Equation 2a).

##### Fatiguing isometric

Parameters *α*_*A*_*, α*_*Km*_*, α*_*τ1*_, and *τ*_*fat*_ were identified from the fatiguing isometric contractions from one testing session. One fatiguing stimulation protocol was applied per subject, at the end of a randomly selected testing session. The knee angle was 90° and the pulse duration was 600 μs. Fifteen pairs of testing (50CFT-12.5VFT) and 195 fatiguing [33CFT (33 Hz)] trains, a total of 225 trains were applied as follows: 1 pair of testing trains followed by 13 fatiguing trains and then repeating the 15 trains 15 times. All train durations were 1 second. The 50CFT and 12.5VFT in the first pair were each followed by a 10 second rest. All remaining inter-train rests were 1 second. The 33CFT and this duty cycle were chosen because both have been proven effective to fatigue the quadriceps within 10 minutes with minimal discomfort to the participants [10]. The 50CFT-12.5VFT pairs, applied after every 13 fatiguing trains, generated the forces used for identification of the isometric fatigue model parameters [10]. The 15 sets of *A*_*90*_*, K*_*m*_*,* and *τ*_*1*_ parameters, derived by minimizing the error between the forces measured for each pair of testing trains and the forces predicted by the force-motion model (Equation 2), were compared to the 15 sets of *A*_*90*_*, K*_*m*_*,* and *τ*_*1*_ parameters predicted by the fatigue model (Equations 4–8) to identify *α*_*A*_*, α*_*Km*_*, α*_*τ1*_, and *τ*_*fat*_ (Figure 2).

#### Non-isometric tests

##### Non-fatigue Non-isometric

Identification of the parameters *V*_*1*_ and *V*_*2*_ in Equation 2b required isovelocity measurements from one testing session during which the exercise dynamometer extended the leg in passive mode. A previous study showed that force-motion model predictions were more accurate when parameters *V*_*1*_ and *V*_*2*_ were identified at 200°/second rather than 125°/second or slower velocities [8]. Therefore, the dynamometer in the current study was set to 150°/second, its maximum velocity in passive mode, and the leg was moved from ~110° to 4°. To obtain only the force due to stimulation, *F*, it was necessary to collect measurements from leg extensions without and with stimulation [8] as the dynamometer extended the leg from 85° to 20°. This range of motion excluded the acceleration and deceleration tails and is within the general non-isometric range of motion of the leg. Four trains were applied, one per leg extension, two 50CFT-12.5VFT pairs with pulse durations of 600 μs and a 10 second rest between each train. Measured forces were compared to the modeled forces (Equation 2) for identification of *V*_*1*_*,* and *V*_*2*_.

Identification of parameters *L/I* and *F*_*M*_ (Equation 3) required general non-isometric non-fatiguing measurements immediately before every non-isometric fatiguing session. The leg was released from the dynamometer, a 4.54 kg load was strapped to the ankle, and the leg swung freely. Potentiation trains were applied to the free swinging leg immediately before the general non-isometric measurements. Two pairs of testing trains (2 × 50CFT-12.5VFT), each followed by a 10 second rest, were applied to the free swinging leg, immediately prior to the fatiguing trains in the fatigue protocol. The train duration was set to the time needed for the leg with attached 4.54 kg load to extend to 10-15° while the thigh was stimulated with a 50CFT at the pulse duration of interest. Measured and modeled angles and angular velocities were compared for every non-isometric session to identify not only the values for *L/I* and *F*_*M*_ (Equation 3), but also to identify the initial, non-fatigue force-motion model parameters, *A*_*90,0*_*, K*_*m1,0,*_ and *τ*_*1,0*_, for the fatigue model (Equations 4–8), thereby adjusting for day-to-day variability.

Five pulse durations were tested: 170, 200, 250, 400, and 600 μs, one per testing day. Previous studies [4,32] measured the greatest changes in force at pulse durations between 100 μs and 250 μs, at frequencies used in the current study. The minimum pulse duration in the current study was set to 170 μs because shorter pulse durations frequently did not produce sufficient excursion of the leg at the amplitude set for the subject (as described above). The next higher pulse duration was set to 200 μs because the greatest changes in peak force occurred at the lowest pulse durations. This small increase in pulse duration produced at least a 5% increase in peak force, as was observed in the previous studies. The average train durations were 0.64, 0.51, 0.36, 0.29, and 0.24 seconds for the pulse durations: 170, 200, 250, 400, and 600 μs, respectively. The train duration for a given pulse duration was held constant for all pulse frequencies.

##### Fatiguing Non-isometric

Five general non-isometric fatiguing stimulation protocols were applied per subject, one per testing day, immediately following the non-fatigue protocol (Figure 1). As with the non-isometric non-fatiguing tests, the leg swung freely with a 4.54 kg load strapped to the ankle and the same five pulse durations were tested: 170, 200, 250, 400, and 600 μs. As with the isometric fatiguing protocol fifteen pairs of testing (50CFT-12.5VFT) and 195 fatiguing [33CFT (33 Hz)] trains, a total of 225 trains were applied. The 50CFT and 12.5VFT in the first pair were each followed by a 10 second rest and were used for identification of the initial parameters, *A*_*90,0*_,* K*_*m1,0,*_ and *τ*_*1,0*_, for the fatigue model (Equations 4–8) as was stated in the non-fatiguing non-isometric section. All remaining inter-train rests were 1.2 seconds, the minimum time required for the leg to return to the resting position (80° to 90°) and to manually stop the oscillations with one’s hands. The train duration remained constant during each fatigue protocol, that is, all 225 trains for a specific pulse duration test had the same train duration.

Parameters *β*_*A*_ and *β*_*Km*_ were removed from the fatigue model and an equation for *β*_*τ1*_ (Equation 8) was derived during model development from correlations between the fitted *β*_*τ1*_ and other force-motion-fatigue model parameters (Objective 1). Predictions for some subjects improved when all three *β* parameters were set to 0. Therefore, values for *β*_*A*_*, β*_*Km*_*,* and *β*_*τ1*_ were estimated separately through optimizations where predictions from the fatigue model, containing just one *β* per optimization, either *β*_*A*_*, β*_*Km*_*,* or *β*_*τ1*_, were fit to the fatigue measurements to determine if one or more *β* parameters could be eliminated. Preliminary results suggested that *β*_*A*_ and *β*_*Km*_ could be removed from the fatigue model. The remaining *β*_*τ1*_ was initially identified by optimizing the fit between the fatigue model values and the angle and angular velocity fatigue measurements for the 170, 200, and 600 μs pulse duration tests. This fitted *β*_*τ1*_ was used in the correlations to derive an equation for *β*_*τ1*_.

### Prediction of outcome measures –experimental data from both the current and previous study

Predicted angular excursion, joint torque due to stimulation, angular velocity, and power (torque (N-m) × angular velocity (rad/s)) were compared over time and under different pulse duration and load conditions. Two pulse durations from the current study and two loads from our previous study [16] were used for the comparisons. From the previous study 4.54 kg and 9.08 kg were chosen. The 4.54 kg was selected because this was used in the current study for all pulse durations and the 9.08 kg was selected because this was the upper limit. From the current study, the pulse durations 600 μs and 170 μs were chosen because these were the lower and upper limits tested. A higher pulse amplitude was required in the current study than in the previous study to extend the leg to ~15° at the lowest pulse duration, 170 μs.

### Statistical analysis

To validate the model, the predictive accuracy of the model was determined by analysis of the linear regression coefficient of determination (r^2^, Objective 2). For each subject and each pulse duration in the current study (170, 200, 250, 400, and 600 μs) or applied load in the previous study [16] (0, 1.82, 4.54, 6.36, and 9.08 kg), the dependent variable was the predicted, and the independent variable was the measured, angular excursion or angular velocity. Both a fixed slope of unity and a y-intercept of zero were used. Ideally, if the predictive accuracy of the model were 100%, then the linear regression r^2^ would be unity. Differences in the subject-averaged r^2^ values between the different pulse durations or applied loads, both for angular velocity and excursion were determined using repeated measures ANOVAs followed by Tukey post hoc tests. A two-factor test was used for the subjects tested in the current study where the independent variables were pulse duration (170, 200, 250, 400, and 600 μs, non-isometric and isometric) and type of subject (development and validation). A one-factor test was used for the subjects tested in the previous study where the independent variable was load (0, 1.82, 4.54, 6.36, and 9.08 kg). In all cases the dependent variable was the r^2^-value.

To present outcome predictions (Objective 3), differences in predicted angular excursion, torque time integral (TTI), joint torque at maximum power, angular velocity at maximum power, and maximum power due to stimulation of the quadriceps were determined using two-factor repeated measures ANOVAs followed by Tukey post hoc tests. The independent variables for the two-factor ANOVAs were pulse duration (170, 200, 250, 400, and 600 μs; measured in the current study) or load (0, 1.82, 4.54, 6.36, and 9.08 kg; measured in the previous study [16]) and contraction number (the first 33CFT and the average of the last seven 33CFTs). The 33 Hz train was chosen because it was used to fatigue the muscle and was the middle frequency train, between the 50 Hz and 12.5 Hz trains. The last seven trains were averaged because the torque-time and angle-time curves typically varied more at the end of the fatigue protocol than at the beginning. Additionally, at the beginning of the fatigue protocol there was a 10 second rest just prior to the first 33CFT, whereas only 1.2 seconds separated the remaining trains in the fatigue protocol. The shorter rest time resulted in somewhat increased variability in the starting position and velocity before each contraction. Because the fatigue curve was at steady state when the last set of 33CFTs was applied, the average of the last half of that set adequately represented the last train. The dependent variables were predicted angular excursion, TTI, joint torque at maximum power, angular velocity at maximum power, and maximum power, all due to stimulation. The predicted joint torque was computed by multiplying the force predicted by the force-motion-fatigue model by the moment arm (L) from the knee joint center of rotation to the center of the load applied just proximal to the ankle. In all cases p < 0.05 was considered significant.

## Results

### Modifications to the fatigue model (objective 1)

**Figure 3:**
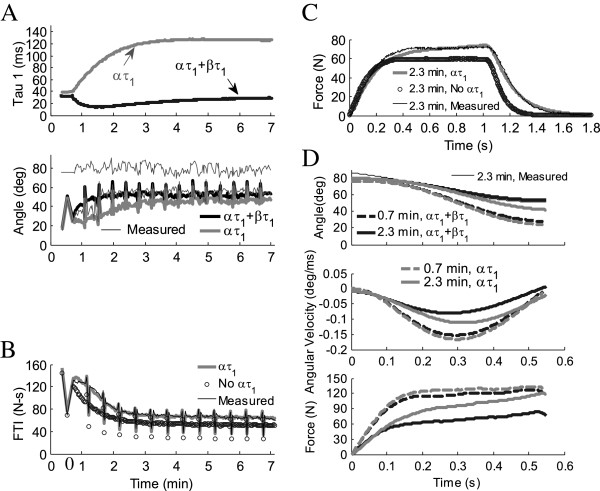
**Effects of fatigue model parameters *α***_***τ1***_**and *β***_***τ1***_**on isometric and non-isometric contractions in one subject where *β***_***τ1***_**was higher than average.** (**A**) Addition of *β*_*τ1*_(dθ/dt) to *α*_*τ1*_ in Equation 8 brings force relaxation time constant, τ_1_, closer to pre-fatigue value (top), resulting in non-isometric predictions with a faster rate of fatigue (bottom). Thin solid black lines are measured values, just prior to (80°) and at end of extension. (**B**) For isometric contractions, removal of *α*_*τ1*_ in Equation 8 keeps τ_1_ constant at pre-fatigue value, resulting in isometric predictions with a faster rate of fatigue (FTI =force time integral). (**C**) A single isometric contraction shows that when *α*_*τ1*_ is included in isometric Equation 8, progressively slower twitch relaxation times increase the force of contraction. (**D**) Single non-isometric contractions show that addition of *β*_*τ1*_(dθ/dt) to Equation 8 partially negates the effect of *α*_*τ1*_. In A. and B. pairs of 50CFT and 12.5VFT testing trains were followed by 13x33CFT fatiguing trains. Contractions in (**C**) and (**D**) occurred at 0.7 min (dashed line) and 2.3 min (solid line). Non-isometric: 4.54 kg load, 250 μs pulse duration. Isometric: 600 μs pulse duration. Initial force-motion model parameters: *A*_*90*_ = 2.10 N/ms, *K*_*m*_ = 3.52e-01, *τ*_*1*_ = 36.1 ms, *τ*_*2*_ = 52.1 ms, *τ*_*c*_ = 20 ms, *R*_*0*_ = 2, *a* = −4.49e-004 deg^-2^, *b* = 3.44e-02 deg^-1^, *V*_*1*_ = 3.71e-01 N/deg^2^, *V*_*2*_ = 2.29e-02 deg^-1^, *L/I* = 9.85 kg^-1^m^-1^, *F*_*M*_ = 247.5 N. Fatigue model parameters: *τ*_*fat*_ = 99.4 s, *α*_*A*_ = −4.03e-07 ms^-2^, *α*_*Km*_ = −1.36e-08 ms^-1^N^-1^, *α*_*τ1*_ = 2.93e-05 N^-1^, *β*_*τ1*_ = 8.54e-04 ms deg^-1^N^-1^.

Complete data sets were collected on 25 subjects. Preliminary regression analyses of predictions of fatigue using the force-motion-fatigue model from the previous study [16], which used equations for *β*_*A*_*, β*_*Km*_*,* and *β*_*τ1*_ from the fatigue model (Equations 4–8), showed that although this model accounted for most of the variance in most subjects, predictions for some subjects improved when all three *β* parameters were set to 0 (not shown). Preliminary results suggested that inclusion of parameter *β*_*τ1*_ alone, without *β*_*A*_ or *β*_*Km*_, in the fatigue model could account for fatigue in all the subjects. Angular velocity multiplied by *β*_*τ1*_ (Equation 8) reduced the impact of fatigue model parameter *α*_*τ1*_ on the force relaxation time constant τ_1_. Parameter *α*_*τ1*_ accounts for the increase in τ_1_ that occurs during isometric fatigue, but in some subjects, parameter τ_1_ changed less during non-isometric fatigue than during isometric fatigue (Figure 3 shows an extreme case). Applying this new fatigue model to measurements from our previous study [16] confirmed that *β*_*A*_ and *β*_*Km*_ were not needed in the fatigue model to predict non-isometric fatigue.

The parameter *β*_*τ1*_ could be expressed as a function of parameters in the non-isometric force and isometric fatigue models. This is shown in Equation 9:

(9) $$\beta_{\tau_{1}}=8.5\times 10^{-11}\times \frac{A_{90,0}^{1.6}\times F_{M}^{0.5}\times \tau_{1,0,\mathrm{iso}}^{3.5}\times \alpha_{\tau_{1}}^{1.3}\times \tau_{\mathrm{fat}}^{0.9}}{V_{1}{}^{0.4}}\text{,}$$

where *A*_*90,0*_*, F*_*M*_*,* and *V*_*1*_ are non-fatigue force-motion model parameter values from the day of the non-isometric fatigue session of interest, *τ*_*1,0,iso*_ is the non-fatigue force-motion model parameter value from the isometric fatigue session, and *α*_*τ1*_ and *τ*_*fat*_ are fatigue model parameter values from the isometric fatigue session. The equation for *β*_*τ1*_ (Equation 9) in the current study is different from the equation in the previous study (Equation 8a) (11) because in the previous study three *β* parameters, *β*_*A*_*, β*_*Km*_*,* and *β*_*τ1*_, were used in the fatigue model. All three were identified simultaneously when fitting the fatigue model (Equations 4–8) predictions to the fatigue measurements to obtain the fitted *β* values used in the correlations to derive the equations for *β*. In the current study only one *β* parameter, *β*_*τ1*_ (Equation 8), was used and identified when fitting the fatigue model predictions to the measurements, therefore the fitted *β*_*τ1*_ in the current study was different from that in the previous study. Because *β*_*τ1*_ could be estimated from equation 9, non-isometric fatigue measurements were not needed to predict non-isometric fatigue.

### Predictions of fatigue validated the model (objective 2)

**Figure 4:**
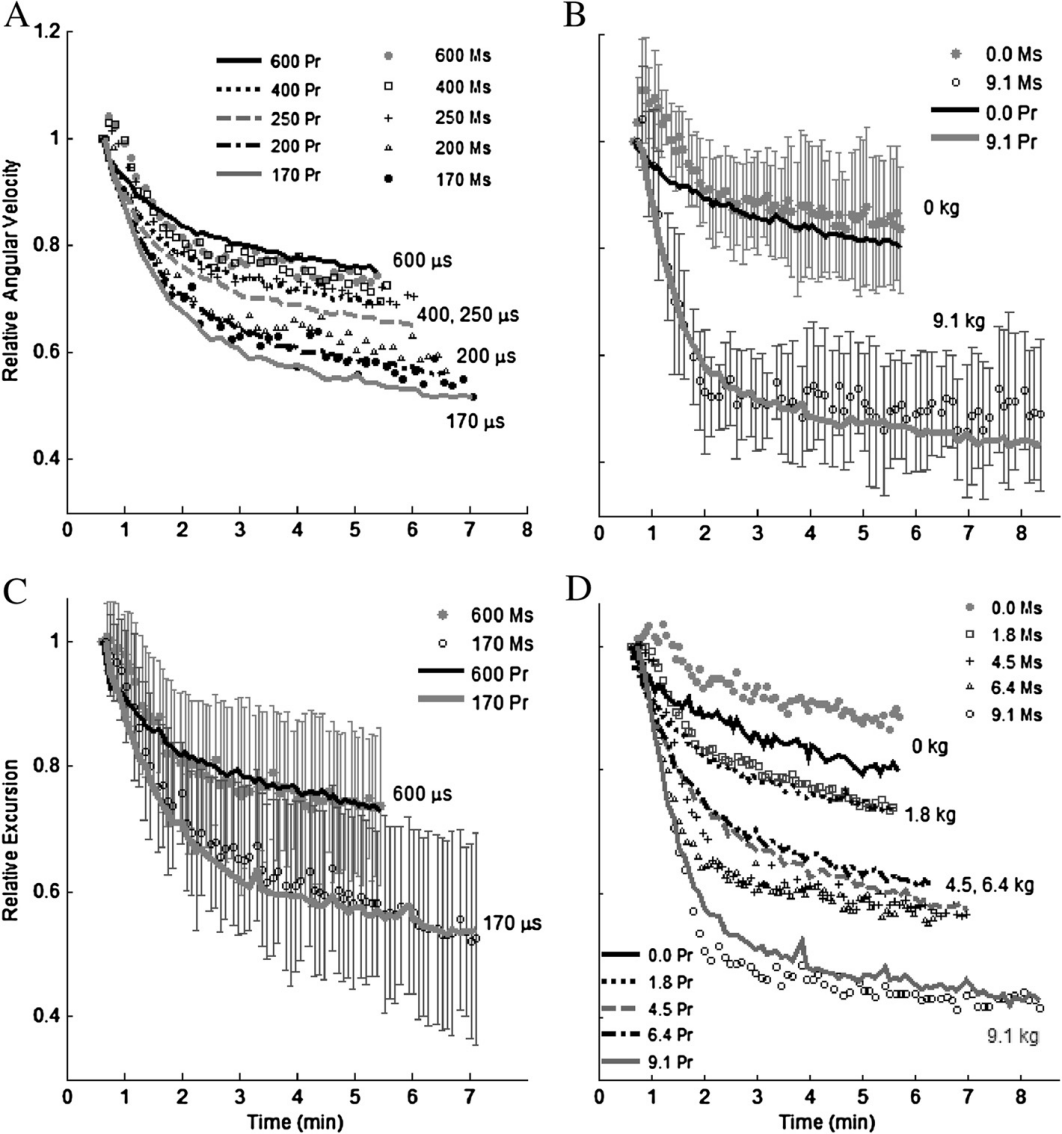
**Measured (Ms) and predicted (Pr) pulse duration (A,C) and load (B,D) dependent reduction in relative angular velocity (A and B±SD) and excursion (C±SD and D) during fatiguing contractions.** Angular excursion is defined as the difference between the initial and final knee angle of a leg extension. The 33CFT contractions shown are normalized to the first contraction. Measurements for (**B**) and (**D**) were collected in our previous study [16]. Predictions are within one standard deviation of measurements, with the exception of the first 1.5 minutes of the 0 kg load. In (**A**) and (**C**) applied load is 4.54 kg and average train durations in (**A**), from shortest to longest pulse duration, are 0.64, 0.51, 0.36, 0.29, and 0.24 seconds. In (**B**) and (**D**) pulse duration is 600 μs and average train durations in (**D**), from highest load to lowest load, are: 0.89, 0.54, 0.51, 0.32, 0.19 seconds. Maintaining a constant excursion necessitated changing the train duration. Only 2 loads and pulse durations are shown in (**B**) and (**C**), respectively, so that the standard deviations could be shown.

Both measured and predicted angular velocity and excursion showed the greatest fatigue at the highest load, shortest pulse duration, and longest train duration (Figure 4, Objective 2). Train duration was a confounding factor, but was consistent across both studies. Predicted velocity- and excursion-time curves were within one standard deviation of measured curves, with the exception of the first 1.5 minutes of the 0 kg load tests (Figure 4B, D).

**Figure 5:**
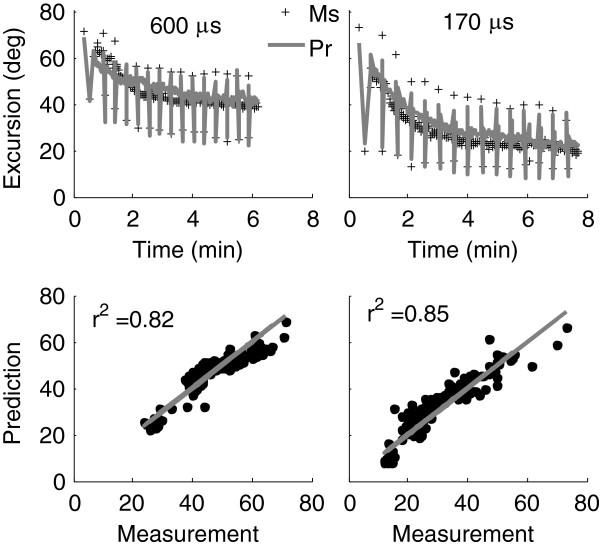
**Predicted (Pr) vs. measured (Ms) angular excursion from one subject for the 600 μs and 170 μs pulse durations.** Applied load was 4.54 kg. Pairs of 50CFT and 12.5VFT testing trains were followed by 13 x 33CFT fatiguing trains (15 sets of 15 contractions for a total of 225 contractions). Row 2 is the linear regression analysis. Both a fixed slope of unity and y-intercept of 0 were used, because this is the ideal relationship between measurement and prediction. Initial force-motion model parameters for the 600 μs and 170 μs pulse durations, respectively, were: *A*_*90*_ = 1.11 and 1.69 N/ms, *K*_*m*_ = 2.75e-01 and 3.60e-01, *τ*_*1*_ = 57.9 and 31.5 ms, *τ*_*2*_ = 59.8 ms, *τ*_*c*_ = 20 ms, *R*_*0*_ = 2, *a* = −3.27e-04 deg^-2^, *b* = 4.21e-02 deg^-1^, *V*_*1*_ = 1.56 N/deg^2^, *V*_*2*_ = 4.98e-02 deg^-1^, *L/I* = 22.2 and 5.86 kg^-1^m^-1^, *F*_*M*_ = 86.3 and 254.7 N. Fatigue model parameters were: *τ*_*fat*_ = 95.4 s, *α*_*A*_ = −4.02e-07 ms^-2^, *α*_*Km*_ = −7.34e-08 ms^-1^N^-1^, *α*_*τ1*_ = 4.17e-05 N^-1^, *β*_*τ1*_ = 4.60e-05 and 1.53e-04 ms deg^-1^N^-1^.

**Figure 6:**
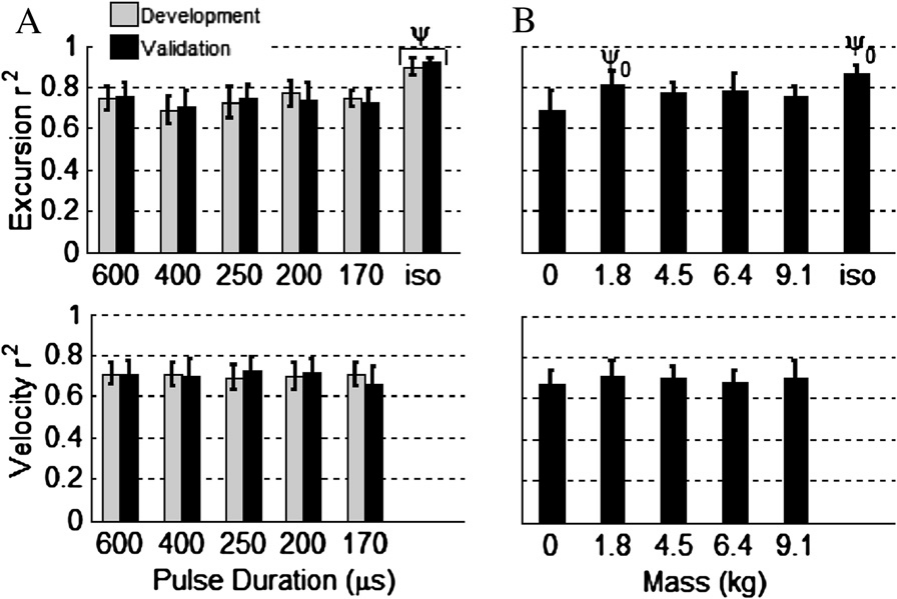
**Average linear regression coefficients of determination (r**^**2**^**; ± 95% confidence limit) for predicted versus measured angular excursion and velocity of all contractions (50CFT, 12.5VFT and 33CFT).** The non-isometric force-motion-fatigue model accounted for 66-81% of the variability in fatigue during general non-isometric leg extensions. Isometric force-time integral at 90° (iso) is shown for comparison. In **A**. n of gray bars = 13 model development subjects (note that only 170, 200, and 600 μs were used for model development) and n of black bars = 12 model validation subjects. Applied load was 4.54 kg. In **B**. n = 10 model validation subjects (pulse duration = 600 μs). ψ_0_ - compared to 0 kg (0.001≤p<0.05); ψ - compared to all 5 pulse durations (0.0001≤p< 0.02). Because the greatest potentiation occurred during the 0 kg load tests and because the force-motion-fatigue model does not include a term for potentiation, predicted excursions and velocities were lower than the measured values for 0 kg.

Comparison of predictions to measurements through linear regression analyses (Figure 5) indicated that the new non-isometric force-motion-fatigue model accounted for between 66% and 77% of the variability in non-isometric fatigue for different clinically relevant pulse durations (170, 200, 250, 400, or 600 μs) with 4.54 kg applied to the ankle (Figure 6A). Predictions of measurements from our previous study [16] indicated that the new model also explained between 67% and 81% of the variability in non-isometric fatigue for different applied loads (0, 1.82, 4.54, 6.36, or 9.08 kg) when stimulating with 600 μs pulses (Figure 6B). Recall that the model development measurements were collected only in the current study and only at 170, 200, and 600 μs. All other measurements were used only for model validation. The predictions for the isometric measurements exceeded those for the non-isometric measurements (0.0001<p< 0.02, Figure 6B), accounting for >85% of the variability in isometric fatigue.

### Outcome measures that can be predicted and compared (objective 3)

**Figure 7:**
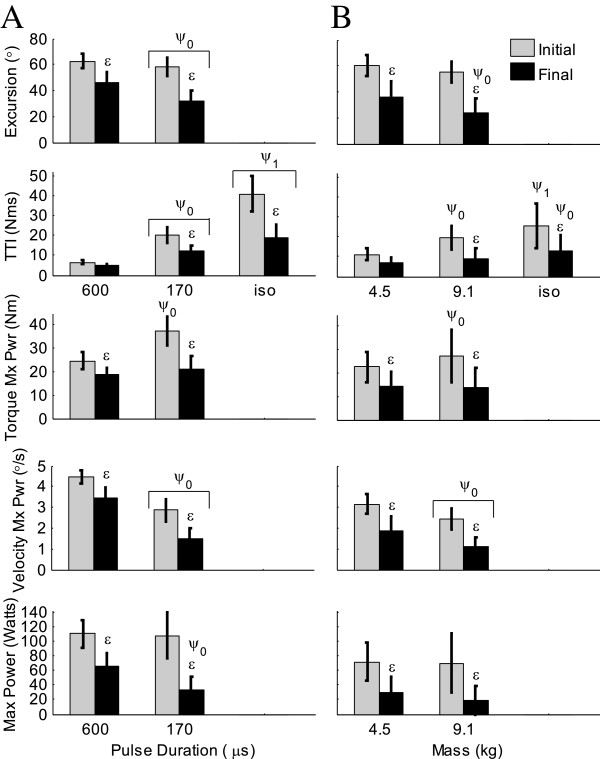
**Predicted angular excursion, torque time integral (TTI), joint torque at maximum power, angular velocity at maximum power, and maximum power, all due to stimulation (mean ± SD; n = 25 (A) and 10 (B) subjects; 33CFT).** Note that initial maximum power was not significantly different between the two loads or between the two pulse durations, but velocity was lowest at the highest load and shortest pulse duration. Isometric TTI (iso; 90°) is shown for comparison. Gray bars = first 33CFT; black bars = average of last half of last set of 33CFTs. Average train durations for 600 μs, 170 μs, 4.5 kg, 9.1 kg, and isometric (600 μs pulse duration) were: 0.24, 0.64, 0.51, 0.89, and 1.00 seconds, respectively. ε – compared to first contraction (p<0.0001); ψ_1_ – compared to 4.5 kg and 9.1 kg (0.0001≤ p≤0.02), or to 600 μs and 170 μs (p<0.0001); ψ_0_ – compared to 4.5 kg (0.0001≤ p≤ 0.03) or to 600 μs (0.0001≤ p≤ 0.02).

Torque at the knee due to stimulation of the quadriceps cannot be measured directly during general non-isometric leg extensions because the leg is not attached to any device that might resist its natural motion. However, this torque can be predicted by our force-motion-fatigue model. In this way, angular excursion, joint torque, angular velocity, and power due to stimulation can be compared over time and under different conditions (Figure 7, Objective 3). The predicted dependent variables showed significant fatigue (contraction number as the independent variable) at both loads and both pulse durations. With applied load or pulse duration as the independent variable, differences between the two applied loads or two pulse durations were not always significant. The predicted initial maximum power was not significantly different between the two loads or between the two pulse durations. The predicted angular velocity at maximum power was significantly less at the highest load and lowest pulse duration, while the predicted initial joint torque at maximum power was significantly greater at the highest load and lowest pulse duration. The initial angular excursion at 170 μs pulse duration was significantly less than at 600 μs (Figure 7A). Keep in mind that the train duration was set such that the 50CFT, not necessarily the 33CFT, produced the maximum excursion at each pulse duration or load.

## Discussion

The key findings in the current study were that

(a) pulse duration was not explicitly needed in the fatigue model; its effects on fatigue were captured by its effects on force,

(b) two of three *β* parameters could be eliminated from our previous fatigue model without loss of predictive value with current and previous data sets,

(c) the remaining *β* parameter is expressed completely as a function of values already measured, so, effectively, no additional parameters were added to the fatigue model,

(d) the new force-motion-fatigue model accounted for 66-77% and 67-81% of the variability in the non-isometric measurements from the current and previous study, respectively, and

(e) the model can be used to compare the power, angular velocity, angular excursion, and joint torque due to stimulation produced during fatiguing non-isometric contractions under different testing conditions.

The fatigue model was simplified by eliminating the parameters *β*_*A*_ and *β*_*Km*_ from the fatigue model and generating a new equation for *β*_*τ1*_ (Equation 9) as a function of existing force-motion-fatigue model parameters. Because *β*_*τ1*_ was multiplied by negative angular velocity, the *β*_*τ1*_ term reduced the effect of *α*_*τ1*_, bringing *τ*_*1*_ closer to its pre-fatigue value (Figure 3). In some subjects the difference between the pre-fatigue and fatigue twitch relaxation times was minimal during non-isometric contractions. For some subjects, the twitch relaxation time during non-isometric fatiguing contractions was less than during isometric fatiguing contractions.

Non-isometric fatigue measurements were not needed to predict non-isometric fatigue. In total, all but 5 parameters (*A*_*90*_*, K*_*m*_*, τ*_*1*_, *L/I* and *F*_*M*_), from both the force and fatigue models were identified from measurements collected during one testing session. The remaining 5 parameters were identified from pre-fatigue general non-isometric leg extension measurements from each non-isometric fatigue testing session.

The predictive ability of our new non-isometric force-motion-fatigue model (0.66 <= r^2^ <= 0.77 for pulse duration and 0.67 <= r^2^ <= 0.81 for applied load) tended to be higher than that of our previous non-isometric force-motion-fatigue model (0.56 < r^2^ <= 0.76 for applied load) [16], though lower than that of our isometric (r^2^ >0.85) force-fatigue model (Figure 6). The predictions in the current study for the measurements collected in the previous non-isometric modeling study [16] tended to be more accurate than those in the previous study because 1) the baseline angle or velocity for every stimulation train (contraction) delivered during the fatigue protocol on a given day in the current study was the initial value before the first train in the fatigue protocol, whereas in the previous study the baseline was an average of the initial angles or velocities before each of the 225 trains, and these sometimes deviated from the baseline of the resting leg and 2) the time between the last potentiation train and the first train in the fatigue protocol was reduced in the current study compared to the previous study, which reduced the magnitude of force enhancement that often occurred within the first few trains in the fatigue protocol. Insufficient potentiation explains why the measured fatigue tended to be less than the predicted fatigue for the 0 kg load (Figure 4) because the force-motion-fatigue model had no provision for potentiation.

A number of factors may explain why the isometric force-fatigue model accounted for more of the variability in the isometric measurements (Figure 6; 86-92%) than the non-isometric force-motion-fatigue model could account for in the non-isometric measurements. These include the following:

(1) In the isometric case, all model parameters were identified from force measurements at one knee angle, 90°. In the non-isometric case, the force-length relationship model parameters were identified from force measurements at 4 different angles and the isovelocity and free model parameters were identified from angle and angular velocity measurements at the angles between ~85° (resting) and ~12° (nearly full extension).

(2) In the isometric case, the electrode position relative to the nerves and muscles beneath was nearly constant from the beginning to the end of a fatiguing protocol. In the non-isometric case, both the skin and muscles moved as the leg extended, and maximum extension depended on pulse frequency and extent of fatigue, and therefore the amount of movement may have varied from train to train.

(3) In the isometric case, the leg remained in the sagittal plane. In the non-isometric case the leg may have moved out of the sagittal plane as it fatigued.

(4) In the isometric case, the potentiation protocol given just prior to the fatigue protocol minimized the force enhancement that often occurred during the first few trains in the fatigue protocol. In the non-isometric case, the potentiation protocol was not as effective at reducing the force enhancement that occurred with the shortest train durations and highest velocities, perhaps due to the continual and rapid change in myofiber or myofibril conformation.

(5) In the isometric case, the initial force immediately before every contraction in the fatigue protocol was the same. In the non-isometric case, the initial angle and angular velocity was not always identical because we manually stopped and released the leg after each fatiguing extension, allowing for some human error.

To reach the desired excursion, as pulse duration decreased, train duration increased; as applied load increased, train duration increased. Train duration was therefore a confounding factor in our results, interacting with pulse duration and applied load (Figure 4). Taken together, the results of both studies suggest that the higher the duty cycle of the train, the greater the fatigue (constant rest time between trains: 1.2 and 1.3 seconds). This has been observed by others [35]. It may seem that holding the train duration the same across all pulse durations (or loads) would have led to a clearer interpretation of the measurements, but then maximum excursion would not have been constant across trials. Both the 12.5 Hz VFT and 50 Hz CFT trains were required to identify the pre-fatigue force-motion model parameters. Considering that the leg was free to move, the maximum train duration was limited by the highest frequency train at the longest pulse duration. Holding the train durations constant would have resulted in significantly different angular excursions among pulse durations, thus creating a different confounding factor. Additionally, our objective was to validate fatigue predictions from excursion and velocity measurements. Using the same pre-fatigue excursion (at 50 Hz) for every pulse duration provided the largest range of excursion between the pre-fatigue and final fatigue measurements.

Comparing initial and final outcome measures in response to different independent variables, such as applied load or pulse duration, could help a therapist determine which stimulation parameters are most desirable for the patient and task. If higher joint torque is required (e.g. to strengthen the muscles), then a pulse duration of 170 μs is preferable to 600 μs (see Figure 7). On the other hand, if maintaining the highest level of power over the greatest length of time is the goal, then a pulse duration of 600 μs is preferable to 170 μs. There was no significant difference in the initial maximum power between the two pulse durations however, the final maximum power for the 170 μs was less than that for 600 μs.

The isometric force model has been shown to perform equally well for both able-bodied and SCI subjects, requiring only minor modifications to the parameter identification procedures for the SCI subjects [28,36]. The new non-isometric force-motion-fatigue model, also validated to account for different loads per activated muscle (which could occur if atrophy progresses), may be equally robust, where similar minor modifications to the parameter identification procedures would pertain to this model. The maximum force generating ability of the muscles could be estimated from peak twitch force measurements as described by Ding, et al. (2005) [36]. The stimulation amplitude could be set as described in the current study, but would not exceed a level consistent with 50% of the force generating ability of the muscles. The isometric experimental protocol and identification of the isometric force model parameters could be similar to that described by Ding, et al. (2005) [36]. The non-isometric experimental protocol could be similar to that described in the current study, but the pulse frequencies would be as described by Ding, et al. (2005) [36]. The model parameters are subject specific, identified by fitting the model to the experimental measurements obtained from one testing session; therefore the current procedure for identifying these model parameters may require only minor modifications for the non-isometric force-motion-fatigue model to predict fatigue in SCI subjects. Spastic measurements would be excluded.

Our model has the potential to help physical therapists design stimulation protocols for patients in rehabilitation programs and to help researchers improve the task performance of FES systems [19,32,37-39]. The isometric force-fatigue model was extensively validated to account for the effect of different pulse frequencies and patterns on fatigue [10,20]. The non-isometric force-motion-fatigue model has been validated to account for different applied loads and pulse durations, and these have resulted in a validation of different train duty cycles. From these model validations we learned that frequency, pulse pattern, pulse duration, and applied load are not explicitly needed in the fatigue model. Their effects on fatigue can be captured by their effects on force. Therefore, the non-isometric force-motion-fatigue model should be able to predict unique combinations of stimulation parameters for different subjects, such that each subject can achieve a desired outcome, such as maintaining a functional level of power for a useful period of time (e.g. Figure 7). The non-isometric force-motion model [39] and the isometric fatigue model [40] have been used in a similar manner in other studies. The force-motion-fatigue model, with all model parameters identified for the task, could either mathematically test combinations of stimulation parameters until the desired outcome is obtained, or could be fit to an experimental force or trajectory (using an optimization algorithm) to generate optimal stimulation patterns that yield the force or trajectory for the desired length of time (see Maladen, et al. [39]).

Because this non-isometric force-motion-fatigue model would be capable of generating subject-specific and task-specific stimulation patterns that can maintain a desired force and motion for a desired length of time into the future, it has the potential for use as a feed forward model in FES systems [41]. If a system either does not use a feed forward model or requires more immediate real time output, then this model could be used to test the performance of the system prior to patient use. The model could generate a series of task-specific optimal stimulation patterns, and these patterns could be compared to the real time FES system selections to optimize the system.

Because able-bodied subjects were tested in our study, there is a small chance that volitional contractions occurred during stimulation. However, it is unlikely that volitional contractions, if present, substantively affected our results. The force-motion-fatigue model has been shown to successfully predict fatigue in response to different frequencies and pulse patterns for numerous subjects over many years [10,27,36,40]. This indicates that the signal-to-noise ratio has been high, where here the stimulated contractions correspond to the signal and the volitional contractions correspond to the noise. In all cases, several testing sessions were performed on each subject, and each session was separated by 48 hours.

## Conclusion

Pulse duration was not explicitly needed in the fatigue model; its effects on fatigue were captured by its effects on force. The non-isometric force-motion-fatigue model from our previous study [16] was simplified to predict non-isometric fatigue both at different applied loads and at different pulse durations. Parameters *β*_*A*_ and *β*_*Km*_ in the previous version of the fatigue model were eliminated and a new equation for the parameter *β*_*τ1*_ was derived. The *β*_*τ1*_ was solely a function of existing model parameters; therefore measurements of non-isometric fatigue are not needed to predict non-isometric fatigue. From 66% to 77% of the variability in the non-isometric measurements for different pulse durations was explained by the new force-motion-fatigue model. This new non-isometric force-motion-fatigue model can be used to predict angular excursion, angular velocity, joint torque, or power due to stimulation at different time intervals during repetitive contractions. This could assist with rehabilitation exercises and with the design and testing of new FES control systems.

## Appendix

### Derivation of the equation of motion

**Figure 8:**
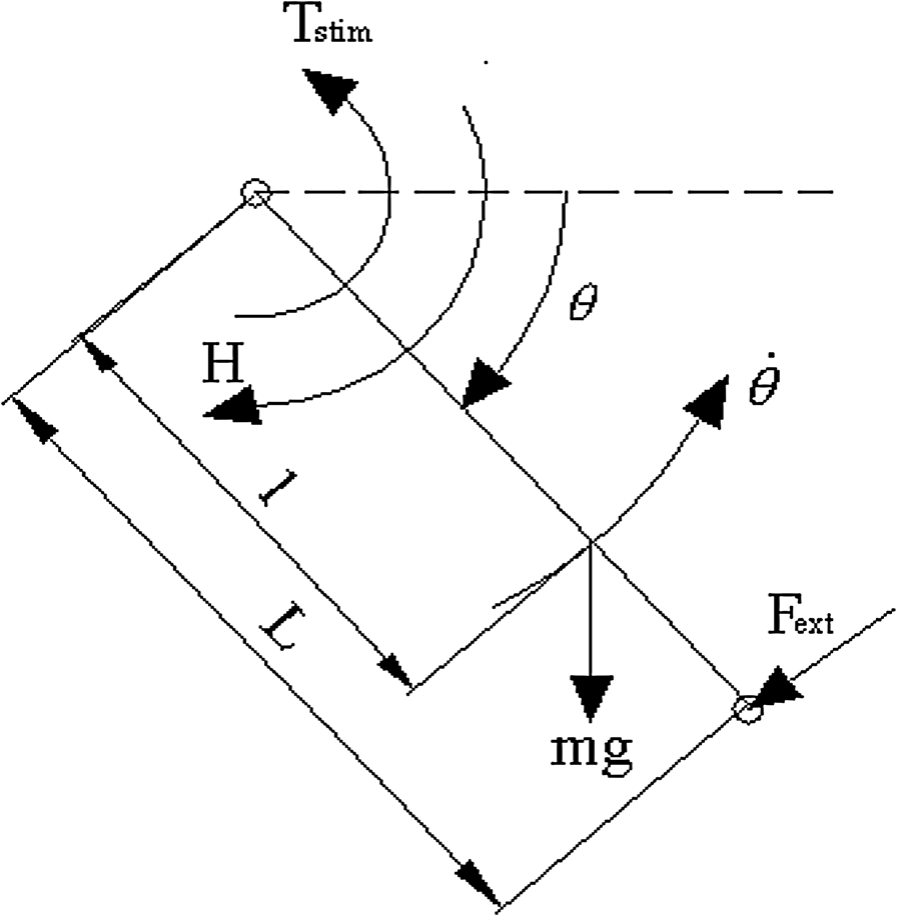
**Schematic representation of the leg, modeled as a rigid body.***L* is the distance from the center of the knee joint to either the center of the calf pad of the Biodex dynamometer knee attachment or the center of the load applied just proximal to the ankle when the leg is not attached to the dynamometer (just proximal to the malleoli but distal to the prominent calf musculature), *l* is the distance from the center of the knee joint to the center of mass of the tibia, *T*_*stim*_ is the torque at the knee due to stimulation, *F*_*ext*_ is the either the force measured by the Biodex dynamometer (*F*_*Bio*_) if the leg is attached to the dynamometer (the resistance that the force dynamometer exerts against the ankle to maintain a constant angular velocity) or the component of the applied ankle weight (*F*_*load*_) that resists the contractile force of the quadriceps if the leg is swinging free, mg is the weight of the leg below the knee and the foot, and *H* is the resistance moment to knee extension due to the visco-elasticity of the structures at the knee.

As described by Perumal, et al [8] the instantaneous moment about the knee center of rotation was derived from the free body diagram of the leg shown in Figure 8.

The equation of motion derived from the free body diagram for isovelocity extensions is:

(10) $$F_{\mathrm{Bio}}L=-\mathrm{mg}l\cos\left(\theta \right)-H+T_{\mathrm{stim}}$$

where *F*_*ext*_ = *F*_*Bio*_ is the force component of the torque measured by the Biodex dynamometer and

(10a) $$T_{\mathrm{stim}}=\mathrm{FL}$$

Thus,

(10b) $$F=F_{\mathrm{Bio}}+\frac{\mathrm{mgl}}{L}\cos\left(\theta \right)+\frac{H}{L}$$

where *F* is the force just proximal to the malleoli exerted by the quadriceps through the knee joint in response to stimulation. It is defined as the instantaneous force near the ankle due to stimulation in Table 1. Previous passive force measurements on healthy subjects showed that (see Perumal, et al [8]):

(10c) $$\frac{H}{L}=R\cos\left(\theta \right)$$

where *R* is an intermediate variable.

Letting

(10d) $$F_{M}=\frac{\mathrm{mgl}}{L}+R$$

and substituting equation 10d into 10b yields

(10e) $$F=F_{\mathrm{Bio}}+F_{M}\cos\left(\theta \right)$$

where *F*_*M*_ is obtained by fitting the function *F*_*M*_ cos(*θ*) to force data collected during passive leg extensions where the quadriceps are relaxed and the dynamometer alone extends the leg.

The equation of motion for the general non-isometric leg extensions is:

(11) $$I\frac{d^{2}\theta }{dt^{2}}=F_{\mathrm{load}}L\cos\left(\theta \right)+\mathrm{mgl}\cos\left(\theta \right)+H-\mathrm{FL}$$

(11a) $$\frac{d^{2}\theta }{dt^{2}}=\frac{L}{I}\left\{\left(F_{\mathrm{load}}+F_{M}\right)\cos\left(\theta \right)-F\right\}$$

Angular acceleration is no longer zero. *F*_*ext*_= the component of *F*_*load*_ (applied ankle weight) that resists the contractile force of the quadriceps. The parameter *L/I* is a lumped parameter encompassing more than length and moment of inertia. Previous estimates of *L/I* using anthropometric data revealed differences from the values estimated through optimization [7,8]. The differences may be the result of: 1) identifying *L/I* and *F*_*M*_ simultaneously during optimization and 2) assuming that acceleration and/or applied weight have no effect on the force-motion model (Equation 2), keeping in mind that *F* in the equation of motion is predicted from the force-motion model (Equation 2). Therefore, *L/I* represents a more generalized parameter.

## Competing interests

The authors declare that they have no competing interests.

## Authors' contributions

MSM, ASW, and MLH conceived of the study, participated in its design and coordination, and drafted and edited the manuscript. MSM wrote the software, tested the participants, performed the optimizations, simulations, and statistical analyses. All authors read and approved the final manuscript.

## References

[B1] Sweeney JD Reilly JP Skeletal muscle response to electrical stimulation Applied bioelectricity: from electrical stimulation to electropathology 1998 1 New York: Springer 299 340

[B2] Thomas CK Nelson G Than L Zijdewind I Motor unit activation order during electrically evoked contractions of paralyzed or partially paralyzed muscles Muscle Nerve 2002 25 6 797 804 10.1002/mus.10111 12115967

[B3] Gregory CM Bickel CS Recruitment patterns in human skeletal muscle during electrical stimulation Phys Ther 2005 85 4 358 364 15794706

[B4] Chou LW Binder-Macleod SA The effects of stimulation frequency and fatigue on the force-intensity relationship for human skeletal muscle Clin Neurophysiol 2007 118 6 1387 1396 10.1016/j.clinph.2007.02.028 17466581 PMC1993846

[B5] Westgaard RH de Luca CJ Motor unit substitution in long-duration contractions of the human trapezius muscle J Neurophysiol 1999 82 1 501 504 10400978 10.1152/jn.1999.82.1.501

[B6] Ferrarin M Pedotti A The relationship between electrical stimulus and joint torque: a dynamic model IEEE Trans Rehabil Eng 2000 8 3 342 352 10.1109/86.867876 11001514

[B7] Perumal R Wexler AS Binder-Macleod SA Mathematical model that predicts lower leg motion in response to electrical stimulation J Biomech 2006 39 15 2826 2836 10.1016/j.jbiomech.2005.09.021 16307749

[B8] Perumal R Wexler AS Binder-Macleod SA Development of a mathematical model for predicting electrically elicited quadriceps femoris muscle forces during isovelocity knee joint motion J Neuroeng Rehabil 2008 5 33 10.1186/1743-0003-5-33 19077188 PMC2615438

[B9] Shorten PR O'Callaghan P Davidson JB Soboleva TK A mathematical model of fatigue in skeletal muscle force contraction J Muscle Res Cell Motil 2007 28 6 293 313 10.1007/s10974-007-9125-6 18080210

[B10] Ding J Wexler AS Binder-Macleod SA Mathematical models for fatigue minimization during functional electrical stimulation J Electromyogr Kinesiol 2003 13 6 575 588 10.1016/S1050-6411(03)00102-0 14573372

[B11] Giat Y Mizrahi J Levy M A musculotendon model of the fatigue profiles of paralyzed quadriceps muscle under FES IEEE Trans Biomed Eng 1993 40 7 664 674 10.1109/10.237696 8244427

[B12] Riener R Quintern J Schmidt G Biomechanical model of the human knee evaluated by neuromuscular stimulation J Biomech 1996 29 9 1157 1167 10.1016/0021-9290(96)00012-7 8872272

[B13] Hawkins D Hull ML Muscle force as affected by fatigue: mathematical model and experimental verification J Biomech 1993 26 9 1117 1128 10.1016/S0021-9290(05)80010-7 8408093

[B14] Tang CY Stojanovic B Tsui CP Kojic M Modeling of muscle fatigue using Hill's model Biomed Mater Eng 2005 15 5 341 348 16179754

[B15] Xia T Frey Law LA A theoretical approach for modeling peripheral muscle fatigue and recovery J Biomech 2008 41 14 3046 3052 10.1016/j.jbiomech.2008.07.013 18789445

[B16] Marion MS Wexler AS Hull ML Predicting fatigue during electrically stimulated non-isometric contractions Muscle Nerve 2010 41 6 857 867 10.1002/mus.21603 20229581

[B17] Kesar T Binder-Macleod S Effect of frequency and pulse duration on human muscle fatigue during repetitive electrical stimulation Exp Physiol 2006 91 6 967 976 10.1113/expphysiol.2006.033886 16873456

[B18] Gregory CM Dixon W Bickel CS Impact of varying pulse frequency and duration on muscle torque production and fatigue Muscle Nerve 2007 35 4 504 509 10.1002/mus.20710 17230536

[B19] Chou LW Lee SC Johnston TE Binder-Macleod SA The effectiveness of progressively increasing stimulation frequency and intensity to maintain paralyzed muscle force during repetitive activation in persons with spinal cord injury Arch Phys Med Rehabil 2008 89 5 856 864 10.1016/j.apmr.2007.10.027 18452732 PMC2665255

[B20] Ding J Wexler AS Binder-Macleod SA A predictive fatigue model–I: predicting the effect of stimulation frequency and pattern on fatigue IEEE Trans Neural Syst Rehabil Eng 2002 10 1 48 58 10.1109/TNSRE.2002.1021586 12173739

[B21] Gorgey AS Black CD Elder CP Dudley GA Effects of electrical stimulation parameters on fatigue in skeletal muscle J Orthop Sports Phys Ther 2009 39 9 684 692 19721215 10.2519/jospt.2009.3045

[B22] Wexler AS Ding J Binder-Macleod SA A mathematical model that predicts skeletal muscle force IEEE Trans Biomed Eng 1997 44 5 337 348 10.1109/10.568909 9125818

[B23] Perumal R Wexler AS Ding J Binder-Macleod SA Modeling the length dependence of isometric force in human quadriceps muscles J Biomech 2002 35 7 919 930 10.1016/S0021-9290(02)00049-0 12052394

[B24] Marion MS Wexler AS Hull ML Binder-Macleod SA Predicting the effect of muscle length on fatigue during electrical stimulation Muscle Nerve 2009 40 4 573 581 10.1002/mus.21459 19626673

[B25] Proceedings of the PSOt - a particle swarm optimization toolbox for use with Matlab: Indianapolis 2003 IEEE: Indiana

[B26] Coleman TF Li YY An interior trust region approach for nonlinear minimization subject to bounds SIAM J Optim 1996 6 2 418 445 10.1137/0806023

[B27] Ding J Wexler AS Binder-Macleod SA A predictive model of fatigue in human skeletal muscles J Appl Physiol 2000 89 4 1322 1332 11007565 10.1152/jappl.2000.89.4.1322

[B28] Ding J Chou LW Kesar TM Lee SC Johnston TE Wexler AS Binder-Macleod SA Mathematical model that predicts the force-intensity and force-frequency relationships after spinal cord injuries Muscle Nerve 2007 36 2 214 222 10.1002/mus.20806 17503498 PMC2633444

[B29] Merrill DR Bikson M Jefferys JG Electrical stimulation of excitable tissue: design of efficacious and safe protocols J Neurosci Methods 2005 141 2 171 198 10.1016/j.jneumeth.2004.10.020 15661300

[B30] Dorgan SJ Reilly RB A model for human skin impedance during surface functional neuromuscular stimulation IEEE Trans Rehabil Eng 1999 7 3 341 348 10.1109/86.788470 10498379

[B31] Petrofsky JS Suh HJ Gunda S Prowse M Batt J Interrelationships between body fat and skin blood flow and the current required for electrical stimulation of human muscle Med Eng Phys 2008 30 7 931 936 10.1016/j.medengphy.2007.12.007 18243763

[B32] Kesar T Chou LW Binder-Macleod SA Effects of stimulation frequency versus pulse duration modulation on muscle fatigue J Electromyogr Kinesiol 2008 18 4 662 671 10.1016/j.jelekin.2007.01.001 17317219 PMC2562565

[B33] Krarup C Enhancement and diminution of mechanical tension evoked by staircase and by tetanus in rat muscle J Physiol 1981 311 355 372 7264972 10.1113/jphysiol.1981.sp013589 PMC1275414

[B34] Zhi G Ryder JW Huang J Ding P Chen Y Zhao Y Kamm KE Stull JT Myosin light chain kinase and myosin phosphorylation effect frequency-dependent potentiation of skeletal muscle contraction Proc Natl Acad Sci USA 2005 102 48 17519 17524 10.1073/pnas.0506846102 16299103 PMC1297671

[B35] Packman-Braun R Relationship between functional electrical stimulation duty cycle and fatigue in wrist extensor muscles of patients with hemiparesis Phys Ther 1988 68 1 51 56 3257300 10.1093/ptj/68.1.51

[B36] Ding J Lee SC Johnston TE Wexler AS Scott WB Binder-Macleod SA Mathematical model that predicts isometric muscle forces for individuals with spinal cord injuries Muscle Nerve 2005 31 6 702 712 10.1002/mus.20303 15742371

[B37] Chou LW Kesar TM Binder-Macleod SA Using customized rate-coding and recruitment strategies to maintain forces during repetitive activation of human muscles Phys Ther 2008 88 3 363 375 10.2522/ptj.20070201 18174446 PMC2659407

[B38] Kebaetse MB Turner AE Binder-Macleod SA Effects of stimulation frequencies and patterns on performance of repetitive, nonisometric tasks J Appl Physiol 2002 92 1 109 116 11744649 10.1152/jappl.2002.92.1.109

[B39] Maladen RD Perumal R Wexler AS Binder-Macleod SA Effects of activation pattern on nonisometric human skeletal muscle performance J Appl Physiol 2007 102 5 1985 1991 10.1152/japplphysiol.00729.2006 17272410

[B40] Chou LW Ding J Wexler AS Binder-Macleod SA Predicting optimal electrical stimulation for repetitive human muscle activation J Electromyogr Kinesiol 2005 15 3 300 309 10.1016/j.jelekin.2004.10.002 15763677

[B41] Bobet J Can muscle models improve FES-assisted walking after spinal cord injury? J Electromyogr Kinesiol 1998 8 2 125 132 10.1016/S1050-6411(97)00029-1 9680953

